# Blocking tri-methylguanosine synthase 1 (TGS1) stops anchorage-independent growth of canine sarcomas

**DOI:** 10.1038/s41417-023-00636-9

**Published:** 2023-06-29

**Authors:** Dora Zucko, Kathleen Boris-Lawrie

**Affiliations:** University of Minnesota – Twin Cities, Department of Veterinary and Biomedical Sciences, Saint Paul, MN 55108 USA

**Keywords:** Drug development, Bone cancer

## Abstract

Tri methylguanosine synthase 1 (TGS1) is the enzyme that hyper methylates the hallmark 7-methyl-guanosine cap (m7G-cap) appended to the transcription start site of RNAs. The m7G-cap and the eIF4E-cap binding protein guide canonical cap-dependent translation of mRNAs, whereas hyper methylated cap, m2,2,7G-cap (TMG) lacks adequate eIF4E affinity and licenses entry into a different translation initiation pathway. The potential role for TGS1 and TMG-capped mRNA in neoplastic growth is unknown. Canine sarcoma has high translational value to the human disease. Cumulative downregulation of protein synthesis in osteosarcoma OSCA-40 was achieved cooperatively by siTGS1 and Torin-1. Torin-1 inhibited the proliferation of three canine sarcoma explants in a reversible manner that was eliminated by siRNA-downregulation of TGS1. TGS1 failure prevented the anchorage-independent growth of osteo- and hemangio-sarcomas and curtailed sarcoma recovery from mTOR inhibition. RNA immunoprecipitation studies identified TMG-capped mRNAs encoding TGS1, DHX9 and JUND. TMG-tgs1 transcripts were downregulated by leptomycin B and TGS1 failure was compensated by eIF4E mRNP-dependent tgs1 mRNA translation affected by mTOR. The evidence documents TMG-capped mRNAs are hallmarks of the investigated neoplasms and synergy between TGS1 specialized translation and canonical translation is involved in sarcoma recovery from mTOR inhibition. Therapeutic targeting of TGS1 activity in cancer is ripe for future exploration.

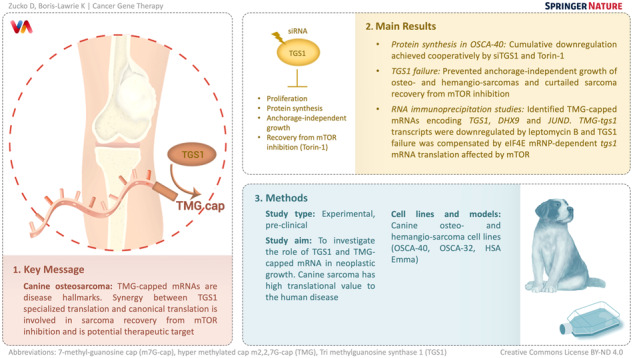

## Introduction

Transactions at the hallmark 7-methyl-guanosine (m7G-cap) are now recognized as a major variable driving the form and function of RNAs generated by RNA polymerase II [1]. Cap is a non-template addition to the transcription start site of nascent RNAs that is co-transcriptionally bound by heterodimeric CBP20/CBP80 complex (CBC). This process is required for productive gene expression and for cell homeostasis.

Cap exchange is the replacement of CBC by alternative cap-binding proteins that sandwich the m7G-structure between two aromatic residues (reviewed in [1]). Cap exchange to eIF4E is necessary for mRNA entry to the eIF4E-dependent translation pathway that is an engine of cell proliferation and cell cycle progression [2, 3]. The translation activity of eIF4E is tightly regulated by the eIF4E binding protein, 4EBP1 [4]. Hypo-phosphorylated 4EBP1 is the allosteric repressor of eIF4E, wherein the hyper phosphorylation of 4EBP1 de represses eIF4E and promptly activates cap-dependent translation. 4EBP1 activity is controlled by the evolutionary conserved kinase, mammalian target of rapamycin (mTOR) and abnormal activity of this regulatory circuit is characteristic of tumor cells [2, 5, 6]. In clinical trials, patients’ tumor growth could be diminished by mTOR inhibitor but on a temporary basis [7, 8]. A new framework posits the repression of eIF4E-dependent translation is rescued by an alternative, specialized translation pathway unaffected by mTOR [9].

Cap exchange to TGS1 is a feature of some transcripts made by RNA polymerase II. Studies have shown TGS1 hyper methylates cap of several selenoprotein mRNAs for eIF4E-independent translation in transformed human cells, setting a precedent to investigate TGS1 activity in sarcoma explants [10]. TGS1 has been shown to hyper methylate HIV-1 incompletely processed transcripts, which antagonize eIF4E-dependent translation [9, 11, 12]. In addition to HIV-1 and selenoprotein mRNAs, few other TMG-capped mRNAs have been identified. The majority of TMG-capped RNA are noncoding small nuclear- and small nucleolar RNAs (sn- and snoRNAs) that are components of ribosomes and spliceosomes and telomerase RNA, hTR [13, 14]. Despite distinct functionalities in the RNA World, each of these RNA species experience m7G-cap hyper methylation that changes the form and directs the function of the mature transcript.

Studies have shown TGS1 binds m7G-cap with high affinity, but is incompatible with TMG-cap [15]. Studies on snoRNPs have shown TGS1 is released from Cap using chaperone activity of CRM1 [16]. The covalent modification of CRM1 by treatment with leptomycin B (LMB) was shown to trap TGS1 on m7G-snoRNPs. Studies have yet-to-determine whether LMB also inhibits biogenesis of TMG-mRNAs.

Whereas TMG-cap fails interaction with TGS1 or other members of the family of m7G-cap-binding proteins, until recently only one vertebrate protein compatible with the TMG-cap structure was known, Snurportin1 [17]. Recent evidence has shown NCBP3/CBP80 is a TMG-cap-binding complex of HIV-1 mRNAs [9] that require RNA helicase A/DHX9 (RHA) for translation [18]. RHA has been shown to bind near the beginning of HIV-1 RNA and tether TGS1 for cap-hyper methylation that licenses translation unaffected by mTOR [9]. Another study identified NCBP3/CBP80-RHA assembles JUND polysomes unaffected by mTOR [19]. RHA also is known to bind near the beginning of the junD RNA and is necessary for JUND translation [18]. Although studies have shown heterodimeric NCBP3/CBP80 and RHA are necessary for junD translation, yet-to-be investigated is the potential role for TGS1 and TMG-cap in junD mRNA unaffected by mTOR [19, 20].

RHA is a ubiquitous RNA binding protein that has been proposed a central node in malignancy [21, 22]. However, controversy exists whether RHA serves in an oncogene- or tumor suppressor role [23, 24]. The *dhx9* gene locus at 1q25 is frequently mutated in breast and prostate neoplasms [25, 26] and RHA overexpression was dubbed a biomarker of malignancies including lung and ovarian cancer [26], hepatocellular carcinoma [27], glioblastoma [28], chemo resistant leukemia [29], and osteosarcoma exhibiting high metastatic ability [30, 31]. RHA binds RNA polymerase II and undergoes co-transcriptional loading to the BRCA1 [31, 32], EGFR [26, 33], MDR1 [34], p16INK4A [35] and p53 RNAs [36, 37]. There are post-transcriptional activities attributed to RHA, including translation that contribute to its enigmatic role in tumorigenesis [18, 21, 22, 38].

Unlike RHA, TGS1 is a relatively underexplored gene (~80 papers reporting on TGS1 on PubMed, accessed on January 26, 2023). Moreover, the data available on The Cancer Genome Atlas (accessed on January 26, 2023) show TGS1 has not yet been reported in bone cancer samples and the levels of expression in cancerous tissues are unknown, as well as whether there is a difference compared to the normal tissues.

Several human and canine sarcomas share clinical, histologic and molecular characteristics [39–42]. Comparative research has yielded therapeutic and diagnostic strategies with translational benefit to canine and human patients [43–45]. OS is a primary malignant bone tumor that occurs at the site of growth plates in the metaphysis of long bones in children and young adults, and canine patients. In people, OS is relatively rare, with 800–1000 cases diagnosed per year, while in dogs the annual incidence exceeds 25,000 cases [41]. OS is highly malignant and over 90% of canine and approximately 30% of pediatric patients succumb to metastatic disease within 2–5 years following diagnosis [39].

OSCA-40 is a malignant canine osteosarcoma explant from a spontaneously developed tumor in the right knee of a 6-year old spayed female Saint Bernard [42]. OSCA-32 is derived from a tumor in the left wrist of a 9-year old spayed female Great Pyrenees [43]. Upon orthotopic implantation into mice, OSCA-40 progressed more quickly and generated more local bone destruction than OSCA-32 [44]. OSCA-40 had higher metastatic propensity, microscopic metastasis to lung and engendered shorter median survival times, but both explants are informative pre-clinical models. Hemangiosarcoma (HSA) [45, 46] is a highly aggressive cancer of endothelial cells sharing the same poor prognosis with OS and provides another comparative model of the human disease. Canine HSA Emma is derived from a metastatic tumor of the brain of a 9-year old spayed female Golden Retriever and closely models all aspects of human angiosarcoma of the breast and viscera, making it an important tool for investigating malignancy [47–49].

Anchorage-independent growth is a hallmark of tumor cells and is associated with their ability to invade and metastasize to other tissues. Yet-to-be-determined are potential roles for TGS1 and TMG-capped mRNAs in neoplastic growth, which could have important implications for understanding the mechanisms of cancer progression and identifying potential targets for therapy. This study investigated whether TMG-capped mRNAs exist and whether TGS1 activity plays a role in promoting anchorage-independent growth in three representative canine sarcomas.

## Materials and methods

### Cell viability assays

OSCA-40, OSCA-32 or HSA Emma were cultured in Dulbecco’s Modified Eagle Medium [DMEM] supplemented with 10% FBS, Primocin and 1% HEPES. Per well of 6-well plates, 5 × 10^5^ cells were seeded for 60 h harvest and 1 × 10^6^ cells were plated for 24 h harvest. Per well of 12-well plates, 2 × 10^5^ to 4 × 10^5^ cells were seeded and medium was supplemented with Torin-1 (100 nM) or LMB (2.5 ng/mL) [50] or both for 24 h. Aliquots of 90 µl were transferred into 96-well plates containing alamarBlue reagent (10 µl, Invitrogen A50100, Carlsbad, CA USA), incubated at 37 °C, 5% CO_2_ for 3 h and relative fluorescent units (RFU) were measured at 560 nm excitation and 590 nm emission wavelengths. The percentage of viable cells in treated and control samples were calculated: Treated RFU / Untreated RFU cell control ×100. Recovery assays were carried out by pelleting aliquots of treated cells, washing with PBS and reseeding in fresh media. The alamarBlue assay was performed after 7-days.

### Anchorage-independent growth assays

Growth in ultra-low attachment (GILA) assays were performed as described [51]. OSCA-40, OSCA-32 or HSA Emma were collected 60 h post-transfection of siRNAs targeting RHA, TGS1 or non-targeting control (siNT) and supplementation with Torin-1 during the final 24 h. Cells were collected, washed in PBS and 1000 cells were seeded per well of 96-well Ultra Low Attachment Plates (Corning 3474, Durham, NC USA) in 100 µl of fresh media. After 5 days, Cell Titer Glo was added to the wells (Promega G755A, Madison, WI USA) according to manufacturer’s instructions and luminescence was detected by an absorbance microplate reader (BioTek Synergy, Santa Clara, CA USA) and interpreted by the same formula as alamarBlue test results. The soft agar protocol was carried out with modifications [52]. Briefly, 1% agarose was diluted with cell culture media in 1:1 ratio and 1 ml of 0.5% agarose was plated into 12 well plates as the base agar layer. Next, 1% agarose was diluted in 1:2 ratio with 1 × 10^4^ cells/ml of DMEM without or with pre-treatments (24 h, Torin-1, LMB, both). One ml of 0.3% agarose + OSCA-40 cells was plated on top of the solidified base layer in triplicates for each treatment, amounting to 2 × 10^4^ cells/well. One ml of cell culture media was added on top (feeding medium). Cells were incubated at 37 °C, 5% CO_2_ for 14 days and the feeding media was exchanged every 3–4 days. Plates were stained using crystal violet and colonies were counted on a Nikon Eclipse Ti-S microscope (Melville, NY USA). Graphpad Prism Version 6 (Graphpad Software Inc., San Diego, CA USA) was used to analyze viability assays and anchorage-independent growth data by Student’s t-test. The mean and standard deviation of the mean (SD) values were always calculated.

### Metabolic labeling

OSCA-40 were supplemented with puromycin (4 mmol, Sigma P7255) for 20 min at 37 °C. Total cell lysates were collected and subjected to WB with indicated antisera. PAGE was carried out on 4 to 20% gradient gels. Proteins were transferred to nitrocellulose, incubated with antiserum and detected by Pierce enhanced chemiluminescence (Thermo Fisher, Waltham, MA USA) on ImageQuant LAS4000 (GE Healthcare Life Sciences, Uppsala Sweden). ImageJ software (NIH, Bethesda, MD USA) performed densitometry. Antibodies used in this study are summarized in Tables S1 and S2.

### RT-qPCR

RNA was prepared in Trizol and aliquots of 0.5 to 1 µg of RNA were subjected to reverse transcription by Omniscript RT in 20–30 µl reactions. Two to 3 µl of each reaction was subjected to qPCR with gene-specific primers (Table S3) and SYBR-Green (Stratagene MX 3005p, San Diego, CA USA). Standard curves were generated on 10^2^–10^8^ copies of DNA amplicons. The mean RNA copies and standard deviation (SD) were calculated and statistical significance between RNA copies and treatment groups was evaluated by Student’s and Welch’s t-tests, respectively.

### RNA interference

Each siRNA (50 nM) (Table S4) was mixed with Lipofectamine 2000 (2.5 µl, Invitrogen) in OPTI-Minimum Essential media (200 µl), incubated for 15 min at RT and dispensed to each well of a 12-well plate seeded with 2 × 10^5^ OSCA-40, OSCA-32 or HSA Emma in 1 mL DMEM. Medium was exchanged eight h post transfection. After 24 h, cells were transfected again, incubated and harvested 60 h after the first transfection. Collected cells were washed in PBS and suspended in a RIPA buffer for 15 min on ice and soluble proteins were collected by centrifugation at 13,400 RPM for 2 min. Pellets suspended in PBS were mixed with TRizol-LS and RNA was extracted per manufacturer instructions (Thermo Fisher).

### Ribosome profiles

Cytosol was extracted from three 10 cm plates of OSCA-40 at 80% confluence. Culture medium was supplemented for 30 min with cycloheximide (CHX) (100 µg/ml, Millipore Sigma O1810) prior to harvest. Cells were washed 3 times with ice cold PBS containing CHX, collected and suspended in ice cold low salt buffer, incubated on ice for 5 min and the lysis buffer was added (Table S5). The samples were decanted to a Dounce homogenizer and subjected to 10 strokes, and centrifuged at 17,000 × *g* for 2 min. The supernates were layered onto 10 ml linear gradients of 10 to 50% sucrose [19] and centrifuged 225,000 × *g* for 2.25 h at 4 °C in a Beckman Coulter Optima XPN-100 Ultracentrifuge, SW41 rotor (Chaska, MN USA). Gradients were fractionated and *A*_254_ measurements were collected on an ISCO system (Lincoln, NB USA). Brandel Peak Trace software was used to process the corresponding profiles and area under the curve.

### Immunoprecipitation (IP)

RNA binding proteins were immune precipitated by magnetic Dynabeads Protein G (30 µl, Invitrogen 10004) conjugated to specific antiserum: rabbit anti-RHA (3 µl, Vaxron PA-001), rabbit anti-eIF4E (4 µl, Sigma E5906) or rabbit anti-IgG (1 × 10^−4^ µl, Sigma I8140) for 30 min at room temperature on a rotating wheel. Beads were collected on a magnet, supernates were removed and beads were washed with NETN and then wash buffer (Table S5) to remove unbound antibody, and incubated with protein lysates for 2–4 h on a rotating wheel at 4 °C. Lysates were isolated from the OSCA-40 suspended in RIPA buffer (Table S5). After incubation for 15 min on ice, soluble proteins were collected by centrifugation at 13,400 RPM for 2 min. Beads were then washed 3 times with cold NETN and 3 times with cold wash buffer. Beads were collected, 10% was reserved for WB and 90% was reserved for RNA extraction.

To isolate TMG-capped RNAs, we modified protocols by Singh [9] and Gribling-Burrer et al. [53]. Briefly, 30 µl aliquots of Dynabeads were incubated with BSA (20 mg/mL) and tRNA (20 mg/mL) for 30 min on a rotating wheel at room temperature; 10 µl of mouse anti-TMG (Millipore Sigma MABE302, Burlington, MA USA) antibody or 1 µl mouse anti-IgG antibody (Sigma I8765, diluted 1:10 000) was added and the beads were incubated at 4 °C overnight on a rotating wheel. Beads were washed once with cold NETN and once with a cold wash buffer. Ten µg of Input RNA was pre-incubated with IgG-coated beads for 30 min and transferred to the TMG-coated beads and incubated at 4 °C for 2–4 h on a rotating wheel. Beads were washed 3 times with ice cold NETN and 3 times with ice cold wash buffer and resuspended in 100 µl water and mixed with 500 µl TRIzol-LS, extracted with chloroform and chloroform-isoamyl alcohol (24:1) and precipitated in isopropanol. RNAs were suspended in 30 µl.

### Statistical analysis

Results are presented as mean with error bars indicating SD. Statistical analysis has been performed using Student’s t-test or Welch’s t-test, as indicated in figure legends. *P*-values of ≤0.05 were considered significant. Experimental replicates were typically *N* = 3 and are specified in the figure legends.

## Results

### Downregulation of TGS1 and RHA cooperatively decrease canine osteosarcoma proliferation and protein synthesis capacity

**Fig. 1:**
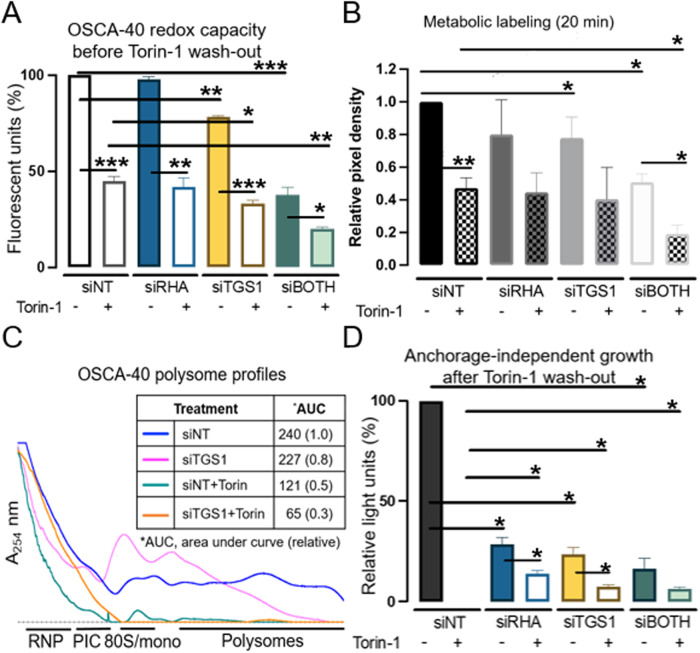
OSCA-40 anchorage-independent growth is abrogated by downregulation of TGS1 and RHA. OSCA-40 cultures transfected with siRNAs for 60 h were supplemented with Torin-1 for the final 24 h. **A** Results of alamarBlue assays on the treated cells have been standardized to siNT control, *N* = 3. **B** Twenty-min metabolic labeling with puromycin and evaluated by Western blot and densitometry. **C** Ribosomal RNA profiles on 10-50% sucrose gradients were detected by spectrophotometry and the area under the curve (AUC) was quantified using Brandel software. Results are representative of 2 experiments. **D** Anchorage-independent growth quantified by Cell titer Glo assays on cells from **C** after wash in PBS. Mean and standard deviation of results from 3 independent experiments are presented. Statistical comparisons have been made using Student’s t-test and are denoted by the horizontal lines (*p ≤ 0.05; **p ≤ 0.01; ***p ≤ 0.001).

To begin to investigate a role for TGS1 and TMG-capped mRNAs in canine sarcoma, we determined the effect of TGS1 downregulation on the proliferation and translation capacity of the OSCA-40 canine osteosarcoma. Titration studies with siRNA targeting tgs1 and dhx9/rha (rha) identified two transfections for 60 h were necessary to diminish TGS1 and RHA by 40 to 60% relative to the non-targeting control siRNA (siNT) (Supplementary Fig. 1A) and Supplementary Fig. 1B. The effect the treatments on OSCA-40 viability was assessed by alamarBlue assays, which detect cellular respiration and redox capacity in proliferating cells by the reduction of resazurin to highly fluorescent resorufin. While the redox capacity was robust in cells treated with siNT or siRHA, it was reduced in cells treated with siTGS1 (siTGS1, *p* ≤ 0.01) and further decreased when siTGS1 were combined with siRHA (siBOTH, *p* ≤ 0.001) (Fig. 1A). As a control, the OSCA-40 cultures were supplemented with the small molecule mTOR inhibitor, Torin-1. Titration experiments identified the minimal dosage of Torin-1 to downregulate P-4EBP1 was similar to that observed in titration studies on human CD4 + T lymphocytes (100 nM) [19]. AlamarBlue assays identified proliferation of OSCA-40 was reduced by 50% in the presence of Torin-1 alone and additionally reduced proliferation of siTGS1- or siBOTH-treated cells (Fig. 1A). OSCA-40 experiencing the wash-out of Torin-1 recovered proliferation similar to control, as shown by alamarBlue assays 72 h later (Supplementary Fig. 2A, white bars). Also, light microscopy of the cultures visualized the differences in cell morphology in the presence and absence of Torin-1 (Supplementary Fig. 2C) and after wash-out and recovery (Supplementary Fig. 2C). To determine relative levels of protein synthesis under these conditions, metabolic labeling with puromycin for 20 min was carried out (Supplementary Fig. 1C). Densitometry results of three replicate experiments showed metabolic labeling was reduced 50% in the presence of Torin-1 (*p* ≤ 0.05), indicating protein synthesis was not abolished by the mTOR inhibitor (Fig. 1B). Treatment with siTGS1 resulted in a 20% decrease in metabolic labeling compared to the treatment with siRHA or siNT control (*p* ≤ 0.05). Notably, the combination of siTGS1 + siRHA was observed to further reduce protein synthesis (*p* ≤ 0.01) and Torin-1 led to another 50% reduction in metabolic labeling, indicating cooperativity between Torin-1 and siTGS1 and siRHA in decreasing protein synthesis (*p* ≤ 0.001). These results were in agreement with results of the proliferation assays (Fig. 1A).

To further investigate OSCA-40 translation activity and the apparent cooperativity between Torin-1 and siTGS1, ribosome profiling was performed. The treatment of OSCA-40 cells with siTGS1 resulted in decreased polysomes and increased 80 S/monosomes relative to the siNT control, indicative of initiation complex formation and impaired loading of ribosomes onto mRNA (Fig. 1C, blue and magenta lines). Torin-1 treatment of OSCA-40 (siNT, green line) resulted in decreased polysomes and 80 S/monosomes, indicating failure to form initiation complexes, as expected. Torin-1 addition to siTGS1 treatment eliminated any residual polysomes and 80 S/monosomes to below the detection limit of the assay (orange line). Analysis of the area under the curve (AUC) documented the reduction in polysomes and 80 S/monosomes observed in the ribosome profiles (Fig. 1C, inset panel) were similar to the reduction in metabolic labeling (Fig. 1B). The assay results agreed that cumulative downregulation of protein synthesis in OSCA-40 cells was achieved through siTGS1 and Torin-1 cooperativity.

Since anchorage-independence is hallmark of neoplastic growth, the treatments were evaluated in assays for anchorage-independent growth. The OSCA-40 treated cells were washed in PBS and cultured in ultra-low attachment plates with fresh medium [51]. After 6 days, anchorage-independent growth was assessed by Cell Titer Glo ATP assays. OSCA-40 anchorage-independent growth was similar between the untreated control and the Torin-1 wash-out cells, reiterating the mTOR inhibitor diminished OSCA-40 proliferation and translation capacity in a reversible manner (Fig. 1D, siNT). The siTGS1 and siRHA treatments significantly diminished OSCA-40 capacity for anchorage-independent growth (*p* ≤ 0.05) (blue and gold bars) and eliminated the recovery from Torin-1 treatment (unfilled bars). The cooperativity between siTGS1 and Torin-1 was recapitulated by siRHA. However, since RHA has been shown necessary for TGS1 activity on HIV-1 mRNA, the results posited that siRHA diminished host TMG-capped mRNA. To determine whether these findings could be generalized to other canine sarcomas, similar investigations were conducted on OSCA-32 and HSA Emma explants.

### TGS1 is necessary for anchorage-independent growth of representative canine sarcomas

**Fig. 2:**
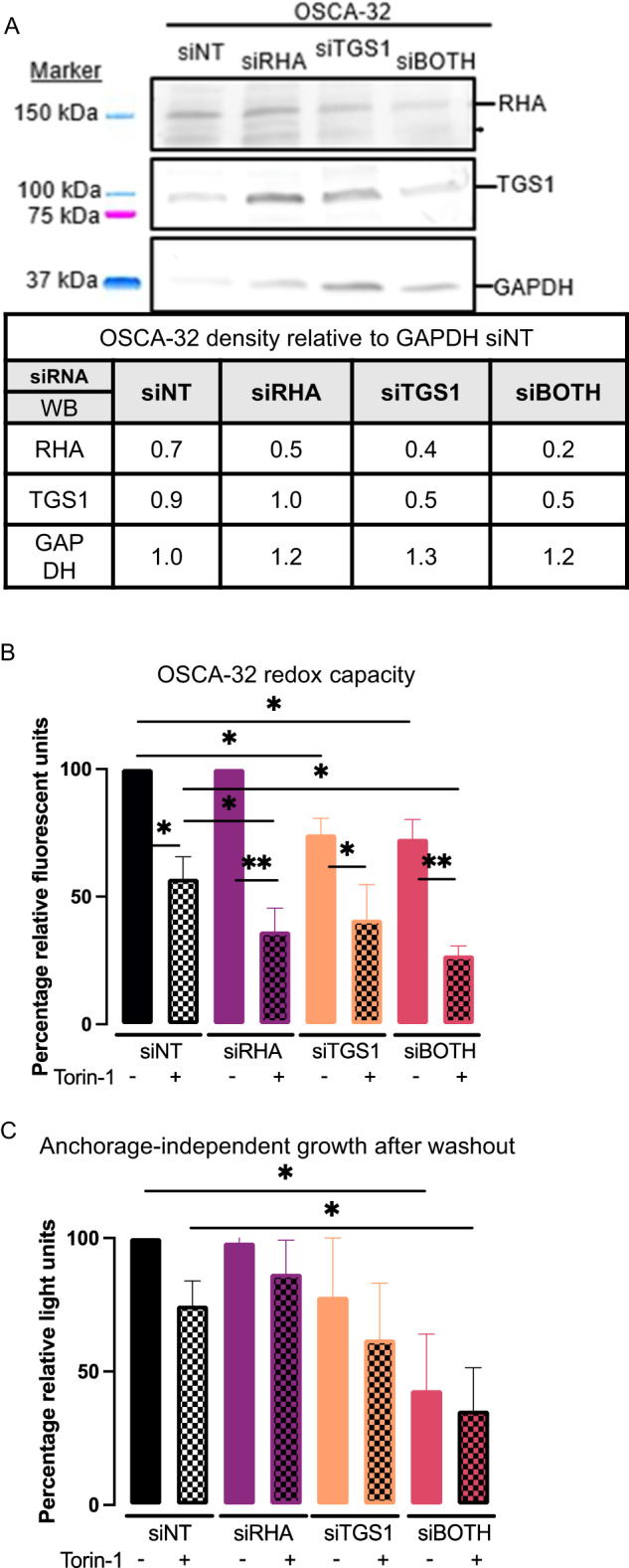
Downregulation of TGS1 and RHA attenuates anchorage-independent growth of OSCA-32. OSCA-32 cultures were treated with the indicated siRNAs for 60 h and supplemented with Torin-1 for the final 24 h. **A** Western blots on siRNA-treated cell lysates with the indicated antisera were quantified by ImageJ and pixel density have been presented relative to GAPDH. **B** Cell proliferation measured by alamarBlue assay are standardized to siNT minus Torin-1 control. Horizontal lines indicate statistical comparisons between the treatment groups. **C** OSCA-40 from **B** were washed in PBS and cultured in ultra-low attachment plates for 6 days without treatments. Results of CellTiter Glo assay have been standardized to siNT. Mean, standard deviation are presented from 3 independent experiments. Horizontal lines report statistical comparisons between treatment groups determined by Student’s t-test. (*p ≤ 0.05; **p ≤ 0.01; ***p ≤ 0.001).

The siRNA treatments, WB regimen and densitometry was carried out on OSCA-32 and identified the specific downregulation of TGS1 or RHA by siTGS1 or siRHA, and that simultaneous siRNA treatment produced the most effective downregulation (Fig. 2A). OSCA-32 redox capacity was significantly decreased by simultaneous siTGS1 and siRHA treatment (siBOTH *p* ≤ 0.05) (Fig. 2B), in agreement with results in OSCA-40 (Fig. 1). Also similar to OSCA-40, Torin-1 treatment resulted in a 50% reduction in OSCA-32 proliferation that was additive with siTGS1 and siBOTH (*p* ≤ 0.05) (Fig. 2B). The results of GILA assays identified OSCA-32 anchorage-independence and recovery after Torin-1 wash-out (Fig. 2C). The anchorage-independence and OSCA-32 recovery from the effects of Torin-1 was not observed in cells experiencing the combination of siTGS1 and siRHA (siBOTH) (Fig. 2C).

In HSA Emma, the siRHA or siTGS1 treatments robustly downregulated RHA or TGS1 proteins (Supplementary Fig. 3A) and Torin-1 treatment, alone and in combination with the siRNA downregulation, significantly reduced redox activity (p ≤ 0.01) (Supplementary Fig. 3B). GILA assays showed HSA Emma anchorage-independent growth was reduced significantly by siTGS1 or siRHA treatment and these cells failed recovery from Torin-1 wash-out of cells (Supplementary Fig. 3C). The results from three representative canine sarcomas agreed that TGS1 and RHA downregulation inhibited anchorage-independent growth. To build on prior research that TGS1 and RHA are necessary for expression of viral TMG-capped mRNA licensed for specialized translation [9], the sarcomas were screened for TMG-capped mRNAs.

### TMG-capped junD, tgs1 and rha mRNAs are present in canine sarcoma

**Fig. 3:**
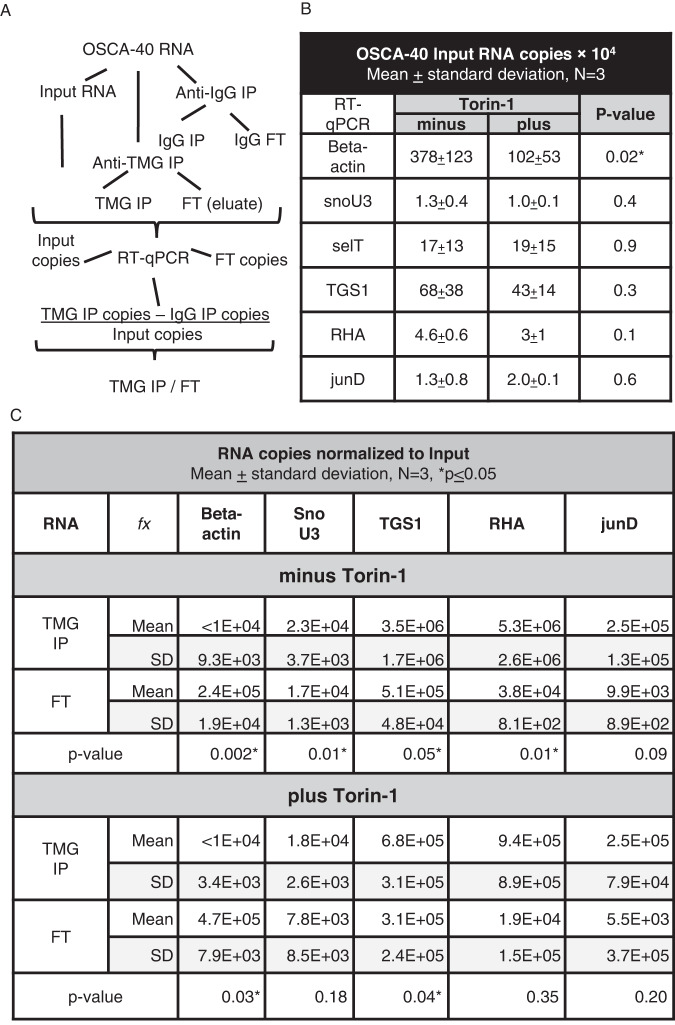
Canine osteosarcoma OSCA-40 express TMG-capped mRNAs. OSCA-40 RNA samples from cells treated with Torin-1 for 24 h or untreated control were precipitated with antiserum to TMG or IgG, followed by RT-qPCR. RNA copies in three independent experiments were determined relative to standard curves. **A** Flow chart of the experiments. **B** Input RNA copies. Significant differences were determined by Student’s t test. **C** RNA copies in TMG IP and FT normalized to Input. Significant differences between IP and FT were determined by Welch’s t-test. *p = ≤0.05.

Building on our previous studies of viral TMG-capped mRNAs in human T-lymphocytes [9], the canine RNAs were collected and subjected to RNA immunoprecipitation with anti-TMG antibody (TMG IP) or IgG control. As depicted in Fig. 3A, OSCA-40 RNA copies were measured between Input RNA, TMG IP and FT samples by RT-qPCR with gene-specific primers and three replicate experiments were carried out. The TMG IP values were subtracted of IgG values and normalized to Input. Regardless of Torin-1 treatment, comparable RNA copies were detected in each Input with the exception of beta-actin (Fig. 3B, *N* = 3). Copies of snoU3, tgs1, rha mRNAs were enriched in the TMG IP compared to copies in the FT (Fig. 3C, *p* ≤ 0.01; 0.05; 0.01, respectively) and junD approached significance (*p* ≤ 0.09). Recapitulating published results in human cells, beta-actin was accumulated in FT (*p* ≤ 0.002), whereas selT enriched in TMG IP (*p* ≤ 0.01) [9, 10]. In the presence of Torin-1, TMG-tgs1 maintained significance, whereas TMG-snoU3, TMG-rha, and TMG-junD did not approach significance (Fig. 3C). TMG IP analysis carried out in HSA Emma recapitulated TMG-snoU3, TMG-tgs1 and TMG-rha transcripts were enriched compared to FT (*p* ≤ 0.005) and junD was below detection in these cells (Supplementary Fig. 4). Taken together, the results documented the presence of TMG-capped mRNAs in canine sarcomas, and suggested experiments were warranted to assess whether siRHA diminished TMG-capped mRNAs.

**Fig. 4:**
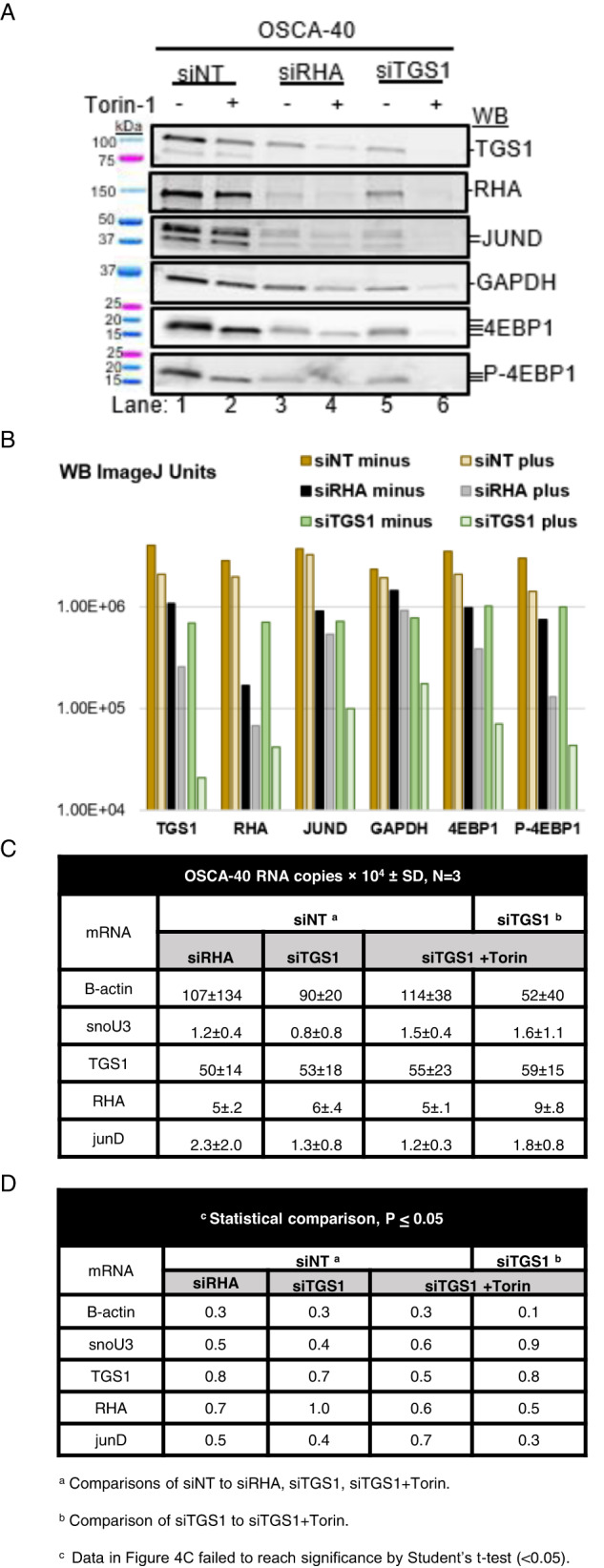
Downregulation of TMG-capped mRNAs by RHA or TGS1 siRNAs and Torin-1. OSCA-40 treated with siRNA for 60 h and cultured without or with Torin-1 for final 24 h were collected. **A** WB carried out on cell lysate with the indicated antisera. **B** Pixel density of WB signals measured by ImageJ have been presented relative to GAPDH siNT on the logarithmic scale. **C** Input RNA copies by RT-qPCR, *N* = 3. **D** Significant differences between Input RNA copies were determined by Student’s t test.

WB and densitometry validated downregulation of TGS1 or RHA, respectively by siTGS1 or siRHA compared to the non-targeting control (siNT) (Fig. 4A, B). Input RNA copies were measured in each of three replicate assays and were not statistically different between the treatment groups (Fig. 4C, D). TMG RNA copies were detected in the siRHA- and siTGS1-treated cells, but the difference between IP and FT no longer reached significance, with the exception of negative control beta-actin was unchanged (Table 1, Welch’s test). TMG-RNA copies were not significantly different between most groups, with the exceptions of TMG-tgs1 RNA copies reduced by siRHA (*p* ≤ 0.01) or by siTGS1+Torin-1. These observations suggested dearth of TMG-tgs1 specialized translation was compensated by m7G-tgs1 mRNA translation affected by the mTOR inhibitor.

**Table 1:** Identification of TMG-mRNA in canine sarcoma OSCA-40.

OSCA-40 RNA copies *N* = 3, TMG IP –IgG normalized to Input*					
**transcript**	**beta-actin**	**snoU3**	**TGS1**	**RHA**	**junD**
**siNT**					
TMG IP	3.40E + 04	2.70E + 04	3.10E + 06	4.30E + 06	1.90E + 05
	2.9E + 04	8.0E + 03	2.1E + 06	3.5E + 06	1.9E + 05
FT	4.1E + 05	8.5E + 03	3.6E + 05	5.2E + 04	9.7E + 03
	1.2E + 05	8.2E + 01	2.3E + 03	1.9E + 04	4.6E + 03
*p*-val	0.002	0.013	0.045	0.054	0.060
**siRHA**					
TMG IP	<1E + 04	1.8E + 04	7.5E + 05	1.5E + 06	3.7E + 05
	2.7E + 03	4.26E + 03	1.07E + 06	1.24E + 06	3.52E + 05
FT	6.8E + 05	1.4E + 04	3.7E + 05	7.0E + 04	1.3E + 04
	1.1E + 05	7.74E + 03	3.27E + 05	3.8E + 04	9.46E + 03
*p*-val	0.001	0.224	0.395	0.328	0.342
**siTGS1**					
TMG IP	5.10E + 04	1.46E + 04	1.53E + 06	1.56E + 06	3.24E + 05
	5.7E + 03	2.7E + 04	5.3E + 05	1.3E + 06	3.3E + 05
FT	4.4E + 05	1.02E + 04	4.40E + 05	4.60E + 04	1.50E + 04
	1.8E + 05	3.6E + 03	1.8E + 05	1.8E + 04	1.3E + 04
*p*-val	0.090	0.390	0.070	0.070	0.090
**siTGS1+Torin-1**					
TMG IP	<1E + 04	1.83E + 04	6.81E + 05	9.44E + 05	2.54E + 05
	1.5E + 03	2.11E + 03	2.50E + 05	7.25E + 05	6.43E + 04
FT	1.66E + 05	1.37E + 04	4.27E + 05	1.18E + 05	1.95E + 04
	1.1E + 05	5.83E + 03	8.27E + 04	1.13E + 05	1.29E + 04
*p*-val	0.050	0.130	0.450	0.230	0.130

^*^Welch’s t-test was performed to determine whether or not there were statistically significant differences in TMG IP RNA copies between the siNT control and siRHA or siTGS1 treatments (Table S1).

### TMG-tgs1 failure unmasks tgs1 mRNAs that are susceptible to mTOR

To further investigate the susceptibility of tgs1 mRNA translation to mTOR, OSCA-40 were treated with Torin-1 in combination with siTGS1 or siRHA in 3 replicate experiments. As expected, WB revealed Torin-1 reduced both the size of 4EBP1 isoforms (4EBP1 WB panel) and the abundance of P-4EBP (P-4EBP1 WB panel) indicative of inhibition of 4EBP1 phosphorylation (Fig. 4A, representative experiment). Densitometry normalized to the GAPDH control (siNT) documented the presence of Torin-1 decreased TGS1 by 50%, and by 90% in combination with siTGS1 (Fig. 4B). The treatment with siRHA alone and in combination with Torin-1 reduced RHA by a factor of 10 (Fig. 4B) and also reduced TGS1. TGS1 was diminished to barely detectable levels by combination with Torin-1 and siRHA and siTGS1 (Fig. 4B), indicative of cooperativity between reducing TGS1 and the mTOR inhibition. The RT-qPCR identified similar Input RNA copies between the treatment groups (Fig. 4C, *N* = 3) indicating the changes in protein levels were not attributable to reduced steady state rha or tgs1 mRNA. The cooperativity between siTGS1+Torin-1 in reducing TGS1 agreed with the global translation reduction observed in the metabolic labeling and ribosome profile experiments (Fig. 1B, C). Notable was that TGS1 was equivalently reduced by Torin-1, siRHA or siTGS1 and there was cooperativity between the treatments (Fig. 4A, B). The results would be explained by *tgs1* transcription products segregated in mRNPs qualified for eIF4E-dependent translation or RHA-mRNPs licensed for specialized translation. Studies in human lymphocytes segregated TMG-capped viral mRNAs licensed for specialized translation in NCBP3/CBP80-RHA mRNPs, whereas downregulation of TMG-cap resulted in accumulation in eIF4E mRNPs susceptible to mTOR inhibition [9].

**Fig. 5:**
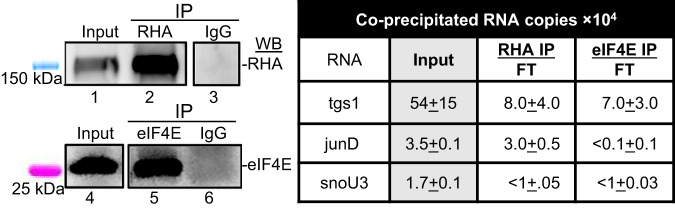
The tgs1 mRNAs are components of eIF4E RNPs and RHA RNPs. OSCA-40 s IPs with antisera to RHA, eIF4E or IgG were analyzed by WB or RT-qPCR. **A** WB with antiserum to RHA or eIF4E on Input, IP and FT samples. **B** Input RNA copies identified by RT-qPCR with gene-specific primers, and the ratio of RNA copies in IP relative to FT. RNA copies in IP were subtracted of IgG IP copies and normalized to Input RNA copies. Mean and standard deviation of three independent experiments.

To test the hypothesis separable populations of tgs1 mRNAs were components of eIF4E-RNPs and NCBP3/CBP80-RHA RNPs, immune precipitates were isolated from OSCA-40 by eIF4E- or RHA-specific antisera or non-immune IgG control and RNA was collected and subjected to RT-qPCR. WB demonstrated robust capture of RHA from Input with RHA-specific antiserum, and robust capture of eIF4E by eIF4E-specific antiserum compared to the IgG negative controls (Fig. 5A, lanes 1-3 and lanes 4-6, respectively). RT-qPCR measured RNA copies in the Input and each IP. The tgs1 transcripts were enriched in both the RHA mRNPs and eIF4E mRNPs, whereas junD copies were only enriched in the RHA mRNP (IP/FT ratio >1) and were less than detectable in eIF4E mRNP (Fig. 5B), recapitulating results in human cells [19]. Also as expected, the noncoding snoU3 RNA failed enrichment in the RHA- or eIF4E-immune complexes and accumulated in FT (IP/FT ratio < 1). The segregated tgs1 mRNA in eIF4E RNPs or RHA RNPs (Fig. 5) agreed with the observation of TMG-tgs1 mRNA for specialized translation and m7G-tgs1 mRNA affected by Torin-1 (Figs. 3, 4).

### LMB downregulated TMG-capped tgs1 transcripts

**Fig. 6:**
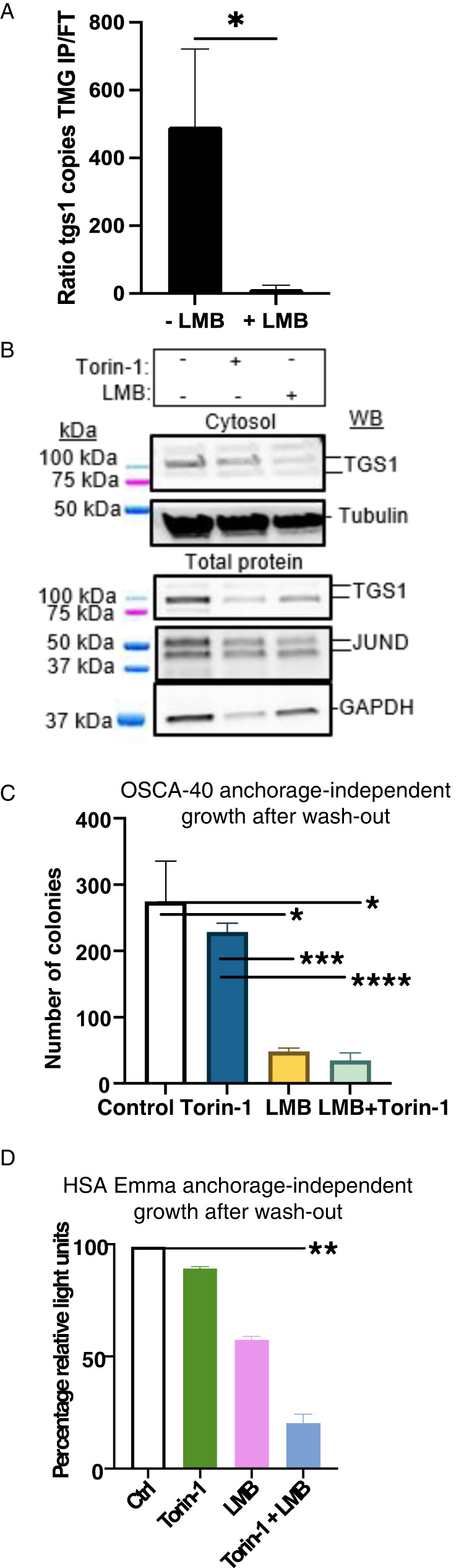
LMB downregulates TMG-tgs1 mRNA, but not tgs1 steady state RNA. OSCA-40 cultures were supplemented with LMB or Torin-1 for 24 h prior to harvest of lysates. **A** Graph presents ratio of IP/FT RNA copies normalized to Input and subtracted of IgG. Mean, standard deviation from three independent experiments. Significant differences were identified by Welch’s t-test (*p ≤ 0.05). **B** WB of cytosol and total protein lysates with indicated antiserum. **C** Anchorage-independent growth of OSCA-40 colonies were collected after 28 days in soft agar. Horizontal lines indicate statistical comparisons. Mean, standard deviation are presented from 3 independent experiments. Significant differences were identified by Student’s t-test (*p ≤ 0.05, **p ≤ 0.01; ***p ≤ 0.001, ****p ≤ 0.0001). **D** HSA Emma growth in ultra-low attachment plates measured by CellTiter Glo ATP assay. Mean, standard deviation are presented from 3 independent experiments with significant between control and Torin-1 + LMB treatment groups (Horizontal line, *p* ≤ 0.05).

Balade et al. identified LMB prevents TGS1 activity on snoRNA independently of nucleocytoplasmic transport of the enzyme [16]. LMB is an antifungal compound that covalently modifies and inactivates the chromosome region maintenance 1 protein (CRM1/Xpo1) that is best known to inhibit nuclear export of cargo proteins that contain a leucine-rich nuclear export sequence, including TGS1 [16]. We considered LMB provides a tool to inhibit TGS1 activity on TMG-tgs1 mRNA independently of nuclear depletion of the enzyme. When OSCA-40 were treated with LMB for 24 h, redox activity reduced significantly (*p* < 0.01–0.001) (Supplementary Fig. 5A), yet cell viability remained similar, as shown by light microscopy (Supplementary Fig. 5B). RNA analysis identified steady state tgs1 mRNA copies were similar between control (untreated) (2.9 ± 1.5 × 10^4^) and LMB-treated cells (2.2 ± 1.1 × 10^4^, *N* = 3 experiments). TMG IP identified TMG-tgs1 transcripts were readily detected in control cells (*p* ≤ 0.05), but were not significantly enriched in the LMB-treated cells (Fig. 6A). These results recapitulated that LMB downregulated TGS1 hyper methylation of snoU3 RNA as identified by Balade et al. [16]. WBs carried out on cytosol and total protein lysates with specific antiserum identified TGS1 was readily detectable (Fig. 6B). TGS1 was readily detectable in the cytosol of cells treated with Torin-1 and diminished in total protein, but LMB diminished TGS1 in the cytosol more than total protein lysates, indicative of nuclear retention by LMB. In total protein lysates, Torin-1 diminished GAPDH, whereas LMB did not. The observation that LMB reduced TGS1 in cytosol samples more than in total protein samples agreed with published results that TGS1 is a substrate for CRM1-dependent nuclear export [16]. Tubulin provided the cytosolic loading control and was not diminished by Torin-1, as shown previously [9]. Next we evaluated OSCA-40 anchorage-independent growth of the LMB-treated or control cells washed in PBS and plated in soft agar. OSCA-40 demonstrated colony formation in soft agar at 4 weeks, as did Torin-1 wash-out cells (Fig. 6C), in agreement with Fig. 1D. However, LMB-wash-out cells and LMB+Torin-1 wash-out cells failed growth in soft agar. Similar experiments were carried out on HSA Emma and identified LMB or Torin-1 reduced proliferation (Supplementary Fig. 5C). When the cells were washed and plated in soft agar, anchorage-independent growth of HSA Emma was similar between control and the Torin-1 or LMB wash-out cells, but failed upon treatment of Torin-1 + LMB (Fig. 6D). The failure of neoplastic growth was attributable at least in part, Torin-1 inhibition of eIF4E-dependent tgs mRNA and LMB downregulation of TMG-tgs1 RNAs for specialized translation.

## Discussion

In this study of canine sarcomas, TMG-tgs1, TMG-junD and TMG-rha mRNAs were identified hallmark of the investigated neoplasms and the blockade of the TMG-mRNA pathway prevented anchorage-independent growth. Notably, the utility of the m7G-tgs1 mRNA as translation templates was compensated by entry into eIF4E-dependent translation pathway affected by mTOR. As a result, the cumulative downregulation of TGS1 required TMG-cap-downregulation and mTOR inhibition and prevented recovery from mTOR inhibitor of all 3 tested sarcomas. In contrast, the m7G-junD or m7G-dhx9 transcripts failed to exhibit utility as translation templates.

Taking into consideration that our findings were made in malignantly transformed canine cells with no non-cancerous comparison, it is not our intention to claim that these observations are specific to cancer. Published studies have documented specialized translation is a constitutive pathway in human lymphocytes that are not transformed [9, 19]. The important open issue that TMG-capped mRNAs are dysregulated in malignancies is well-beyond the scope of this article. Studies are warranted given many reports of RHA and JUND dysregulation in neoplasms and the important role we have identified for TGS1 in recovery from mTOR inhibition. The results confirmed the prediction that TMG-cap is necessary for specialized translation of JUND, resolving the longstanding issue that JUND is unaffected by rapamycin [19, 20] and adding to the notably complex regulation of this AP1 family transcription factor [54].

How does blockade of TMG-tgs mRNA prevent sarcoma recovery from mTOR inhibitor? The data exposed newly appreciated linkage between the eIF4E-dependent and specialized translation mechanisms, as depicted in Supplementary Fig. 5. TMG-mRNPs are licensed for specialized translation unaffected by (upper wheel), whereas m7G-mRNPs require eIF4E activity controlled through mTOR (lower wheel). Experimental results showed the blockade of TGS1 activity (inhibitory treatments) downregulated TMG-tgs1 mRNA, yet TGS1 failure was compensated by m7G-tgs1 mRNA entry to the eIF4E-dependent translation pathway (lower wheel). The significance of linkage between eIF4E-dependent TGS1 translation and TMG-tgs1 mRNA specialized translation is broad. Fouled linkage between the pathways has the potential to phenocopy disease attributed to eIF4E dysregulation. The results suggest therapeutic targeting of TGS1 activity in cancer is ripe for future exploration.

In closing, the dynamic regulation of cap-dependent translation has been an emerging paradigm since the discovery that hyperactive mTOR and eIF4E-dependent translation are hallmarks of tumors [2], and that select mRNAs undergo de-capping accompanied by recapping in a polysome-associated, enzymatic process [55]. The traditional view that exchange of CBC to eIF4E, or another m7G-cap binding protein, supports widespread cap-dependent mRNA translation has been challenged [9, 56]. Now recognized is that TGS1 epigenetic modification of the m7G-cap engenders TMG-capped HIV-1 [9] and herein, host TMG-tgs1 and TMG-junD that are licensed for cap-dependent translation unaffected by mTOR. Blockade of TGS1 hyper methylation of m7G of tgs1 mRNA, and potentially other substrate mRNAs, stops canine sarcoma anchorage-independent growth and recovery from mTOR inhibition.

## Supplementary information

Supplemental material index and Figures

Supplementary tables

## Data Availability

Raw data have been provided. Reagents will be made available upon reasonable request.
