# Supplementary material for: Blocking tri-methylguanosine synthase 1 (TGS1) stops anchorage-independent growth of canine sarcomas
Source: Cancer Gene Ther. 2023 Jun 29;30(9):1274–84. doi: 10.1038/s41417-023-00636-9 (PMC10501901; doi:10.1038/s41417-023-00636-9)
Supplement: Supplementary file 1 — Supplemental material index and Figures [file 41417_2023_636_MOESM1_ESM.pdf]

INDEX

Supplementary Figures and Tables

Blocking tri-methylguanosine synthase 1 (TGS1) stops anchorage-independent growth of canine sarcomas

Supplementary Figure 1: OSCA-40 metabolic labeling is reduced significantly by siRNA downregulation of TGS1 or RHA.

Supplementary Figure 2: Proliferation of OSCA-40 is inhibited by Torin-1.

Supplementary Figure 3: Anchorage-independent growth of HSA Emma is inhibited by TGS1 downregulation by siRNA or Torin-1.

Supplementary Figure 4: TMG-mRNAs are detected in canine hemangiosarcoma Emma.

Supplementary Figure 5: Treatment with LMB irreversibly halts sarcoma proliferation.

Supplementary Figure 6: Synergy between mTOR and TGS1 has been identified for recovery of OSCA-40 from mTOR inhibition.

Table S1: Statistical analysis of data in Table 1.

Table S2: Antibodies used for immunoprecipitation and western blot studies

Table S3: Oligonucleotide sequences

Table S4: Sequences of small interfering RNAs

Table S5: Buffers

Supplementary Fig. 1.

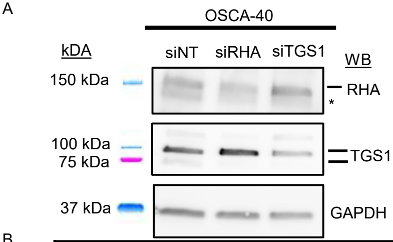

B

Density relative to GAPDH siNT			
siRNA / WB	siNT	siRHA	siTGS1
RHA	1.3	0.7	0.9
TGS1	1.2	1.0	0.7
GAPDH	1.0	1.0	0.9

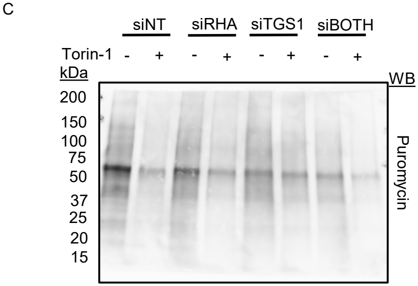

Supplementary Figure 1: OSCA-40 metabolic labeling assay is reduced significantly by siRNA downregulation of TGS1 or RHA. OSCA-40 cells experienced non-targeting siRNA (siNT) or siRNA targeting canine RHA (siRHA) or TGS1 (siTGS) or both siRHA+siTGS1 for 60 h and Torin-1 supplementation for the final 24 h prior to 20 min metabolic labeling with puromycin and WB analysis. **A.** Western blot (WB) of cell lysates with indicated antisera. **B.** Densitometry of WB by ImageJ has been presented relative to GAPDH siNT. **C.** PAGE of equivalent lysates and WB by puromycin antiserum. Densitometry has been presented in Fig. 1A. Representative results of 3 experiments.

Supplementary Fig. 2.

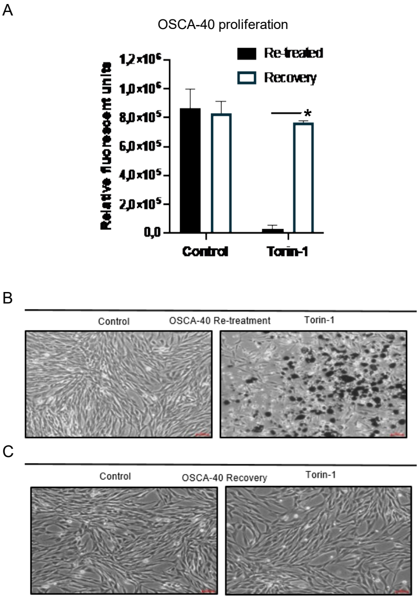

Supplementary Figure 2: OSCA-40 proliferation is inhibited by Torin-1 but recovers after drug is washed out. OSCA-40 were treated with Torin-1 for 72 h, washed in PBS and cultured in fresh medium without Torin-1 (“Recovery”) or with Torin-1 (“Re-treated”) for 7 days in conventional tissue culture dishes. **A.** Results of alamarBlue assay indicate Torin-1 reversibly halts growth of OSCA-1. *, p=<0.05 **B.** OSCA-40 treated with Torin-1 for 72 h, washed in PBS and re-treated with Torin-1 for 72 h were imaged by light microscopy (40× magnification). Cytotoxicity (black) was attributable to prolonged treatment with mTOR inhibitor. **C.** OSCA-40 treated with Torin-1 for 72 h, washed in PBS and cultured without Torin-1 for 72 h were imaged by light microscopy (40× magnification).

Supplementary Fig. 3.

A

HSA Emma density relative to GAPDH siNT				
siRNA / WB	siNT	siRHA	siTGS1	siBOTH
RHA	0.7	0.3	0.4	0.2
TGS1	0.9	0.7	0.5	0.5
GAPDH	1	1.3	1.2	1.1

B

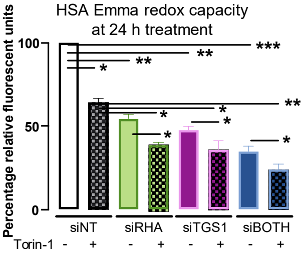

C

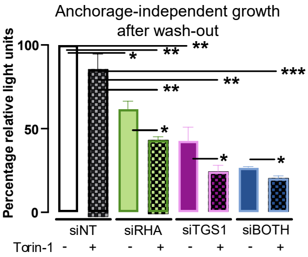

Supplementary Figure 3: Anchorage-independent growth of HSA Emma is inhibited by TGS1 downregulation by siRNA or Torin-1. HSA Emma cultures were treated with siRNAs for 60 h and supplemented with Torin-1 for the final 24 h, followed by WB and growth assays. **A.** WB signals collected by ImageJ have been presented relative to GAPDH. **B.** Treated cells were subjected to alamarBlue assay and results were standardized to siNT. Horizontal lines indicate statistical comparisons between treatment groups determined by Students t-test (* = p $\leq$0.05; ** = p $\leq$0.01; *** = p $\leq$0.001). **C.** Anchorage-independent growth in ultra-low attachment assays was measured after 6 days in CellTiter Glo ATP assays. Mean, standard deviation are presented from 3 independent experiments. Horizontal lines indicate statistical comparisons between treatment groups as in **B**.

Supplementary Fig. 4.

A

HSA Emma Input<br>Input RNA copies ×10^4 ±SD	
Beta-actin	115±95
SnoU3	1.0±0.2
TGS1	72±18
RHA	4.1±0.4
junD	6.5±1

B

TMG IP / FT RNA copies standardized to Input [p-value ≤0.05]						
Transcript		Beta-actin	snoU3	TGS1	RHA	junD
Mean ± SD	TMG IP	<MD	2±0.4 [0.0002]	8±8 [0.007]	35±15 [0.0002]	<MD
	FT	1±0.2	1±0.5	2±1.5	1±0.9	<MD

Supplementary Figure 4: TMG-capped mRNAs are detected in canine hemangiosarcoma Emma. Total RNA isolated from HSA Emma was subjected to IP with TMG antibody or empty control. N=3. **A.** Input RNA copies. **B.** RNA copies in TMG IP and FT. TMG IP normalized to Input and subtracted of IgG control. Significance between TMG IP and FT were determined by Welch's t-test.

Supplementary Fig. 5

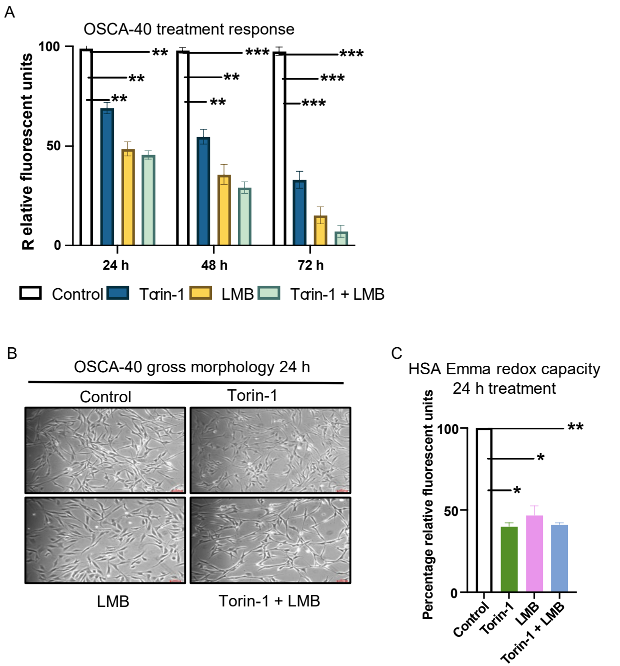

Supplementary Figure 5: Treatment with LMB irreversibly halts sarcoma proliferation. OSCA-40 cultures were supplemented with Torin-1, LMB, Torin-1+LMB or no drug (Control) for 24, 48 or 72 h in triplicate and aliquots were collected for WB or treated with alamarBlue to detect loss of viable cells. **A.** Fluorescence at 560/590 nm was measured at each time point. Data are scaled relative to Control at 24 h. Horizontal lines indicate the comparison between the treatment and the Control for each time point. **B.** OSCA-40 representative light microscopy images at 24 h treatment (40× magnification). **C.** HSA Emma proliferation measured by alamarBlue assay after 24 h treatment. Horizontal lines indicate significance between treatment groups in response to Torin-1, LMB or Torin-1 + LMB, N=3; * = p $\leq$0.05; ** = p $\leq$0.01.

.

Supplementary Fig. 6.

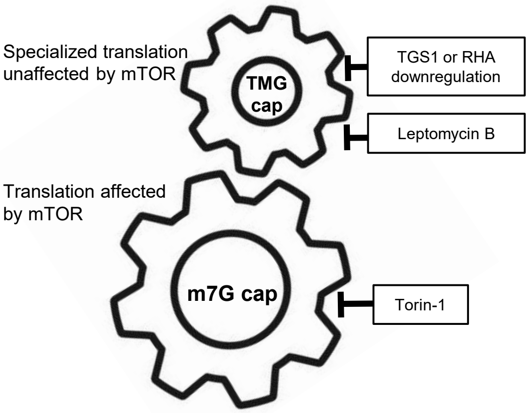

Supplementary Figure 6: Synergy has been identified between mTOR and TGS1 for recovery of OSCA-40 from mTOR inhibition. TGS1 activity is necessary for hyper methylation of select m7G capped mRNAs, which are licensed for specialized translation unaffected by mTOR (top wheel). Failure of TGS1 activity or components of TMG-mRNPs abrogates specialized translation (inhibitory lines). mTOR activity is necessary for eIF4E-dependent translation of m7G-capped mRNAs (bottom wheel) and is inhibited by Torin-1 (inhibitory line). TGS1 downregulation reprograms client mRNAs for canonical translation that is inhibited by Torin-1.
