# Supplementary material for: Blocking tri-methylguanosine synthase 1 (TGS1) stops anchorage-independent growth of canine sarcomas
Source: Cancer Gene Ther. 2023 Jun 29;30(9):1274–84. doi: 10.1038/s41417-023-00636-9 (PMC10501901; doi:10.1038/s41417-023-00636-9)
Supplement: Supplementary file 2 — Supplementary tables [file 41417_2023_636_MOESM2_ESM.docx]

**Table S1. Statistical analysis of data of Table 1.**

TMG-tgs1 and –snoU3 RNA copies are significantly reduced by siRHA.


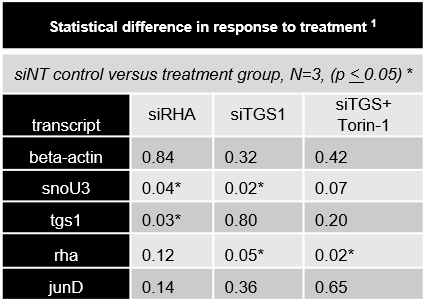


**^1^** Welch's t-test was performed to determine whether or not there was a statistically significant difference in TMG normalized to Input RNA between siNT control and siRHA or siTGS1 treatment.

# Table S2: Antibody reagents.

| **Immunoprecipitation** | **Source** | **Catalog No.** | **Per 30 µl beads** |
| --- | --- | --- | --- |
| DHX9/RHA | Proteintech | 67153-1-Ig | 3 µl |
| eIF4E | Sigma | E5906 | 4 µl |
| TMG | EMD Millipore | MABE302 | 10 µg |
| **Western** | **Source** | **Catalog No.** | **Dilution** |
| DHX9/RHA | Vaxron | PA-001 | 1:50,000 |
| eIF4E | Cell Signaling | #9742S | 1:1,000 |
| 4E-BP1 | Cell Signaling | #9452S | 1:500 |
| P-4E-BP1 | Cell Signaling | #9451S | 1:500 |
| GAPDH | Proteintech | 60004-I-Ig | 1:20,000 |
| JunD | Santa Cruz | SC-74 | 1:1,000 |
| Puromycin | EMD Millipore | MABE343 | 1:20,000 |
| TGS1 | Invitrogen | PA5-82895 | 1:500 |
| Tubulin | Santa Cruz | SC-23948 | 1:1,000 |

**Table S3: Oligonucleotide sequences of primers for quantitative real time PCR.**

| **Name** | **Canine gene** | **Sequence 5’- 3’** |
| --- | --- | --- |
| KB1371 | gapdh sense | CATCAATGACCCCTTCATTGAC |
| KB1372 | gapdh antisense | CGCCCCACTTGATTTTGGA |
| KB2408 | tgs1 sense | CCAGGGCATTTGTGGAGGATC |
| KB2409 | tgs1 antisense | CCTCTTCTTCTGTCGCCTGGT |
| KB2454 | snoU3 sense | TTCTCTGAACGTGTAGAGCACC |
| KB2455 | snoU3 antisense | GATCATCAATGGCTGACGGCAG |
| KB2466 | rha sense | GTACACCCAAGTGGGTCCTG |
| KB2467 | rha antisense | AGTTGTCTGACAAGTGACAGGG |
| KB2472 | tp53 sense | CACCCTTCAGATCCGTGGGC |
| KB2473 | tp53 antisense | GGTGGCTGGAGTGAGCCC |
| KB2476 | beta actin sense | GCATCGTCACCAACTGGG |
| KB2477 | beta actin antisense | ATCTTCTCGCGGTTGGC |
| KB2478 | selenoprotein N sense | AGCTTCATCAGCACTTGGTC |
| KB2479 | selenoprotein N antisense | TGGAGGTGATATCCAAGAAGTAGTT |
| KB2480 | selenoprotein T antisense | CTGGGTACCGCTGGCTTATA |
| KB2449 | selenoprotein T sense | TCCAGATTTGTGTTTCCTGAGG |
| KB2483 | jund sense | CAAGCGCAAGCTGGAGC |
| KB2484 | jund antisense | GTCGGCAGCCGCTGTTGACG |

**Table S4. Small interfering RNA sequences for canine gene downregulation studies.**

| **siRNA** | **Sequence** | **Source** |
| --- | --- | --- |
| dhx9/rha | GAAUGACCUGGGAAGCCAAdTdT | Dharmacon |
| tgs1 | CUGUUACGAUUGAUAGUGAAAGCTC | Integrated DNA Technologies |

**Table S5. Buffers**

RIPA buffer

0.05 M Tris-HCl [pH 8]

0.15 M NaCl

1 mM EDTA

0.25% deoxycholic acid

1% NP40

Add fresh before use: 1× phenylmethylsulphonyl fluoride and 2 mM Dithiothreitol (DTT)

NETN buffer

0.02 M Tris-HCl [pH 7.4]

0.15 M NaCl

0.5% NP40

0.1 mM EDTA

WASH buffer

50 mM Tris-HCl [pH 7.4]

0.15 M NaCl

Polysome lysis buffer is composed of component A and B

Component A: Low salt buffer (LS)

0.02 M Tris-HCl [pH 7.5]

3 mM MgCl_2_

0.01 M NaCl

RNAse-free water

Add fresh before use: 100 ug/mL cycloheximide 5 ul/mL RNAse OUT

Component B: Lysis buffer

0.2 M sucrose

1.2 % Triton X-100

Ratio: 3 volumes LS (prepared) to 1 volume Lysis buffer
